# Immune involvement in neuropsychiatric disorders: Insights from single‐cell transcriptomic studies

**DOI:** 10.1111/pcn.70018

**Published:** 2025-12-27

**Authors:** Tsutomu Takeda, Michihiro Toritsuka, Hiroto Tamakoshi, Nakao Iwata, Manabu Makinodan

**Affiliations:** ^1^ Division of Transformative Psychiatry and Synergistic Research, International Center for Brain Sciences Fujita Health University Aichi Japan; ^2^ Department of Psychiatry Fujita Health University School of Medicine Aichi Japan; ^3^ Department of Psychiatry Nara Medical University School of Medicine Nara Japan; ^4^ Department of Neuropsychiatry Kumamoto University Kumamoto Japan

**Keywords:** immunology and psychiatry, neurological disorders, psychiatric disorders, single‐cell RNA sequencing

## Abstract

Neuropsychiatric disorders pose profound challenges to both research and treatment, largely due to their clinical heterogeneity and the limited understanding of their underlying biological mechanisms. While bulk RNA sequencing (bulk RNA‐seq) has been widely used to study gene expression, it cannot resolve cell‐type‐specific signals or detect rare cellular subpopulations. In contrast, single‐cell RNA sequencing (scRNA‐seq) and single‐nucleus RNA sequencing (snRNA‐seq) have emerged as transformative technologies, enabling transcriptomic profiling at single‐cell resolution. These approaches have revealed immunological alterations across a wide range of disorders. This review introduces recent findings from sc/snRNA‐seq studies of immune‐related mechanisms in psychiatric disorders—including schizophrenia, bipolar disorder, major depressive disorder, autism spectrum disorder, and attention‐deficit/hyperactivity disorder—as well as in neurological conditions such as Alzheimer's disease, Parkinson's disease, dementia with Lewy bodies, multiple sclerosis, and anti‐NMDA receptor encephalitis. While sc/snRNA‐seq overcome averaging effects of bulk RNA‐seq by resolving cell types, these methods still face challenges. We outline a roadmap that integrates bulk RNA‐seq and sc/snRNA‐seq to mitigate the remaining gaps.

Brain science is a particularly challenging field of research in mammals due to the extraordinary complexity of the nervous system.^1^ The human brain, in particular, exhibits advanced structural and functional capabilities, which are believed to contribute to the unique susceptibility of humans to neuropsychiatric disorders.^2^ Although increasing evidence suggests that both psychiatric and neurodegenerative disorders are associated with immune system dysfunction, the complexity of the brain has hindered progress in elucidating their underlying mechanisms. To address these difficulties, researchers employ various methodologies, among which transcriptomic analysis has become increasingly prominent.

Central to transcriptomics is RNA sequencing (RNA‐seq), which utilizes next‐generation sequencing technologies that enable comprehensive profiling of gene expression. Traditionally, RNA‐seq refers to bulk RNA sequencing (bulk RNA‐seq), which captures average gene expression across large populations of cells. Bulk RNA‐seq has advanced our understanding of gene expression, genome reorganization, and splice variants, offering important insights into cellular functions and disease mechanisms.^1^ However, its resolution is inherently limited, as it does not account for the heterogeneity among individual cells within a population. In the brain, the distribution and types of neurons and nonneuronal cells vary across regions, and even cells from morphologically similar populations can show distinct expression profiles due to stochastic variation and microenvironmental influences.^1^, ^3^, ^4^, ^5^ Consequently, bulk RNA‐seq may obscure subtle but biologically meaningful transcriptomic differences (Fig. 1).^6^

**Fig. 1:**
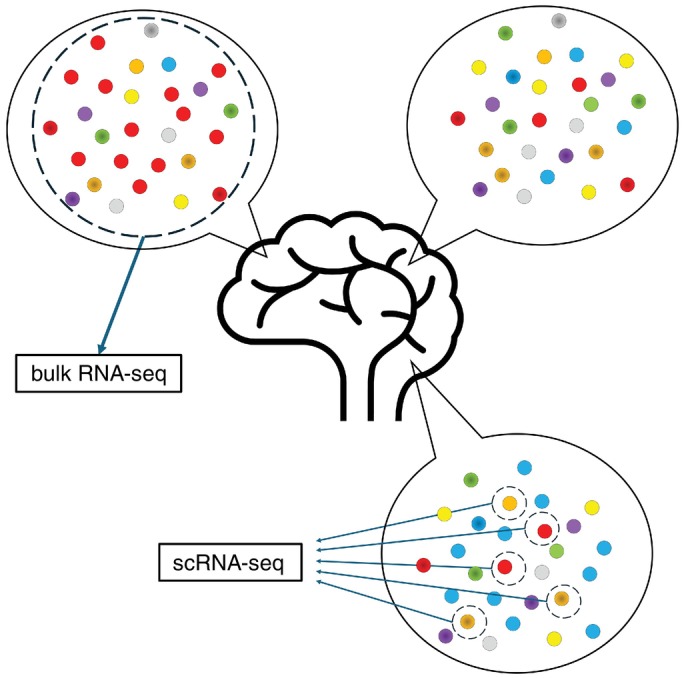
Brain cells differ in type and abundance between neuronal and nonneuronal types depending on the neuroanatomical region, and even cells from supposedly homogenous populations can exhibit different expressions. Bulk RNA‐seq ignores these differences, but scRNA‐seq can analyze each cell individually to detect these differences. RNA‐seq, RNA sequencing; scRNA‐seq, single‐cell RNA sequencing.

To overcome these limitations, single‐cell RNA sequencing (scRNA‐seq) was developed. The primary distinction between scRNA‐seq and bulk RNA‐seq lies in the isolation of individual cells prior to RNA extraction. Various single‐cell isolation techniques have been employed, such as limiting dilution, micromanipulation, flow cytometry, laser‐capture microdissection, and microfluidics (e.g., droplet‐in‐oil, pneumatic membrane valves, and hydrodynamic traps).^1^, ^7^, ^8^ While an exhaustive discussion of these approaches exceeds the scope of this article, an illustrative schematic overview is provided in Figure 2. However, the quality of single‐cell data heavily depends on the ability to isolate viable single units from tissue. Obtaining high‐quality viable brain cells is particularly challenging.^9^, ^10^, ^11^ First, fresh tissue is essential for isolating living cells, but fresh brain samples are difficult to obtain. Second, the brain is a highly interconnected tissue that contains large and complex cells, which further complicates dissociation. To overcome these challenges, single‐nucleus RNA sequencing (snRNA‐seq) has emerged as a powerful alternative. This technique enables the extraction of nuclei from frozen tissue samples—such as those stored in brain banks—and circumvents many of the limitations associated with cell isolation. While both scRNA‐seq and snRNA‐seq enable transcriptomic profiling at the single‐cell level, they differ notably in the origin and composition of the captured RNA.^12^ scRNA‐seq targets whole cells and primarily detects cytoplasmic, mature messenger RNA (mRNA), including spliced variants that are actively translated into proteins. These isoforms can be critical for capturing disease‐relevant expression patterns, including those of clinical relevance. In contrast, snRNA‐seq focuses on nuclear RNA, which contains a higher proportion of unspliced pre‐mRNA. These differences can influence gene quantification and downstream analyses, necessitating careful consideration when interpreting results or comparing datasets generated by the two methods.

**Fig. 2:**
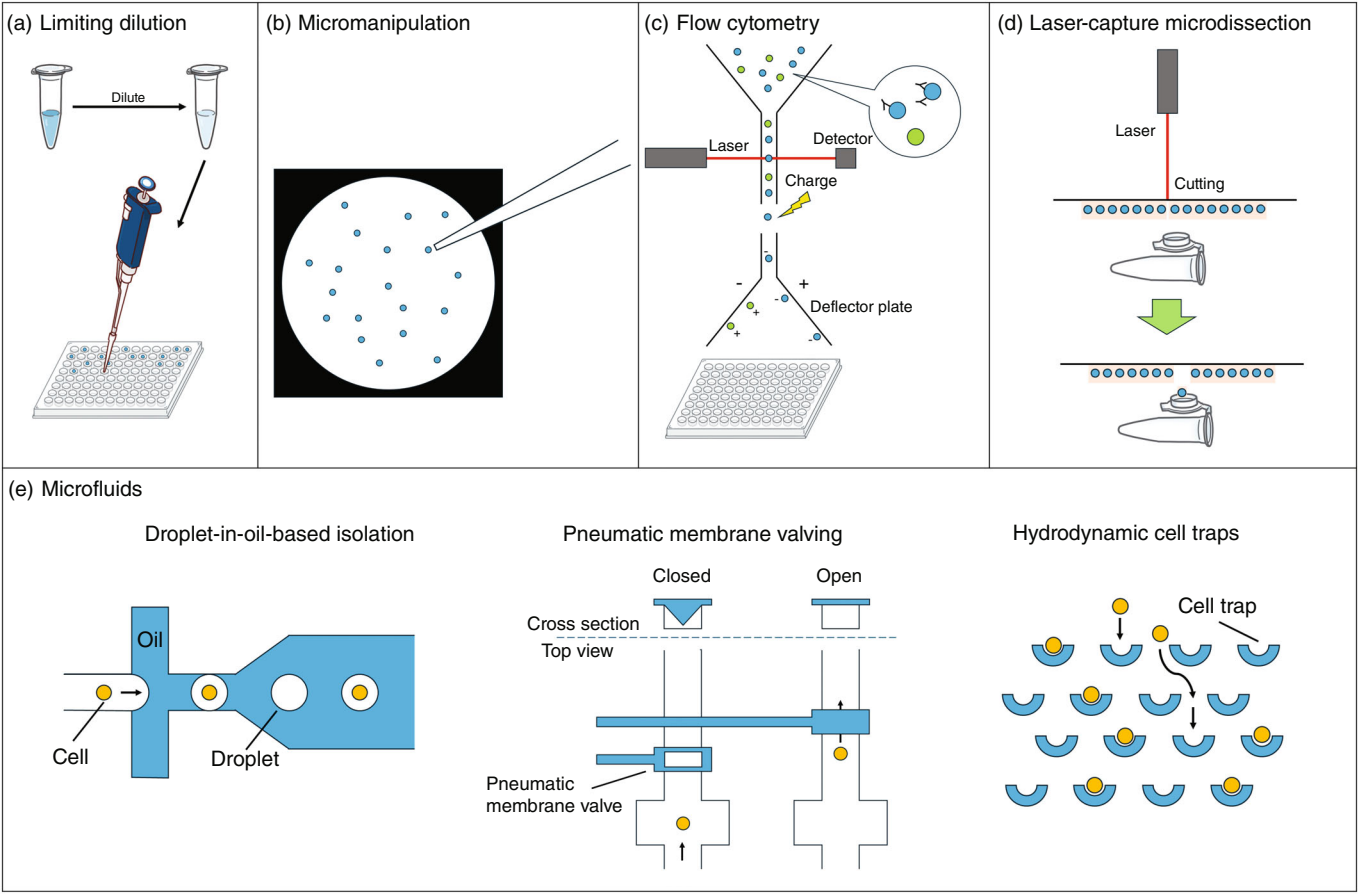
Methods of single‐cell isolation. (a) Limiting dilution. (b) Micromanipulation. (c) Flow cytometry. (d) Laser‐capture microdissection. (e) Microfluidics (droplet‐in‐oil‐based isolation, pneumatic membrane valving, and hydrodynamic cell traps). This figure was created based on the figures by Antunes *et al*., Gross *et al*., and Fang *et al*.^1^, ^7^, ^8^

Importantly, sc/snRNA‐seq enable the detection of subtle transcriptomic alterations that are often obscured in bulk RNA‐seq data. For instance, in a study using human brain tissue, 54 risk genes associated with schizophrenia were identified through scRNA‐seq, two‐thirds of which had not been detected in previous bulk RNA‐seq datasets.^13^ On the other hand, scRNA‐seq has technical limitations. For example, isolating cells with complex morphologies—such as mature neurons or cardiomyocytes—may alter their transcriptomic state.^1^ In contrast, bulk RNA‐seq allows tissue to be homogenized directly in RNA extraction reagents, preserving the native transcriptome. Most sc/snRNA‐seq protocols detect only polyadenylated RNAs, thus excluding noncoding RNAs and reducing transcript detection sensitivity.^1^, ^14^ Furthermore, the small amount of RNA per cell poses challenges for complementary DNA synthesis and amplification.^15^ Nonetheless, sc/snRNA‐seq continue to grow in popularity and has significantly advanced our understanding of cellular diversity, enabling identification of rare or functionally distinct cell types.^3^ Other advanced transcriptomic approaches have also emerged, including spatial transcriptomics, enabling the quantification of gene expression while preserving spatial information within tissue samples, along with RNA deconvolution methods that allow inferring cell‐type composition and cell‐type‐specific expression profiles from bulk RNA‐seq data.^16^, ^17^

sc/snRNA‐seq have been widely applied in the fields of neuroscience and neuropsychiatry and are actively utilized in large‐scale initiatives such as the BRAIN Initiative Cell Census Network (BICCN), the BRAIN Initiative Cell Atlas Network (BICAN), and PsychENCODE. This review highlights recent sc/snRNA‐seq studies that investigate the immunological aspects of major psychiatric disorders—such as schizophrenia, bipolar disorder (BD), major depressive disorder (MDD), and neurodevelopmental disorders—as well as neurological conditions closely related to psychiatry, including various forms of dementia. By focusing on the immune‐related findings uncovered through sc/snRNA‐seq, we aimed to clarify the shared and distinct immunopathological features across these disorders and discuss the implications for future research and therapeutic development.

In summary, bulk RNA‐seq has been indispensable for defining disease‐associated pathways and differential expression at scale, but its averaging of heterogenous populations can obscure cell‐type‐specific signals. By contrast, sc/snRNA‐seq resolve cellular heterogeneity and transient states, providing insights that are invisible to bulk assays.^1^, ^6^, ^16^, ^17^ At the same time, single‐cell approaches remain technically challenging, with issues such as dissociation artifacts, sampling bias, doublets, ambient RNA, and analytical complexity limiting sample size and longitudinal application.^1^, ^12^, ^15^, ^16^, ^17^ These complementary strengths and limitations underscore the need for integrated study designs and cautious interpretation when comparing bulk and single‐cell data.

## Schizophrenia

Schizophrenia is a psychiatric disorder with a global prevalence of approximately 1%. It is characterized by a constellation of symptoms, including delusions, hallucinations, disorganized speech, grossly disorganized or catatonic behavior, and negative symptoms such as diminished emotional expression or avolition. Although its exact etiology remains unclear, it is widely believed to result from a combination of genetic and environmental factors. Increasing evidence also suggests that neuroinflammation and immune dysregulation contribute to its pathogenesis.^18^ For example, one study demonstrated that somatic mutations arising during the early differentiation of brain cells into neurons may play a critical role in the development of schizophrenia, and these mutations are influenced by maternal immune activation.^19^

Bulk RNA‐seq studies have identified consistent immune‐related transcriptional changes in schizophrenia. For example, an analysis of lymphoblastoid cell lines from patients with schizophrenia revealed over 1000 differentially expressed genes (DEGs), many of which are involved in immune responses.^20^ Further meta‐analysis of RNA‐seq and array datasets has highlighted dysregulation of 647 genes involved in immunity.^20^ Postmortem brain studies similarly identified 1146 DEGs, most of them upregulated and one‐third showing aberrant DNA methylation; these genes were enriched in inflammatory and nitric oxide signaling pathways.^21^ Additional bulk RNA‐seq indicates the upregulation of complement component 4A (C4A), the top GWAS‐implicated SCZ disease gene, in the brain with schizophrenia, which has been implicated in the elimination or pruning of synapses.^22^

A growing body of sc/snRNA‐seq evidence also supports the involvement of immune dysregulation in schizophrenia. For instance, an analysis of cultured mesenchymal cells derived from nasal‐turbinate tissue in patients with schizophrenia revealed aberrant expression of immune‐related genes, including *S100A2*, *CCL14*, *IL32*, *HLA‐DRA*, *CCL3*, *CCL4*, *CXCL1*, *CXCL8*, *CXCL10*, *NFKBIA*, *IL1B*, *IL6*, and *TNF*.^23^ These genes are implicated in key immune pathways such as interleukin (IL)‐17 signaling, adaptive immune response, antigen processing and presentation, and gut immunoglobulin A production. Additional findings from scRNA‐seq studies using patient‐derived brain organoids identified an increased proportion of endothelial cells with elevated expression of genes involved in angiogenesis, vascular regulation, and inflammation.^24^ These transcriptional changes were accompanied by structural alterations in vascular architecture and increased endothelial permeability, suggesting a potential role for microvascular dysfunction and impaired blood–brain barrier (BBB) integrity in the pathogenesis of schizophrenia. Recent scRNA‐seq investigations uncovered numerous schizophrenia risk genes that had been missed by bulk RNA‐seq and converged primarily on neuronal/synaptic programs rather than immune dysregulation.^13^, ^25^ As described above, C4A seems to be implicated in the pathobiology of schizophrenia, while snRNA‐seq data in the brain revealed the abnormal C4A‐related pathways in astrocyte and microglia.^26^

These observations collectively suggested that bulk RNA‐seq in schizophrenia consistently indicates broad immune activation—particularly complement signaling, interferon‐related pathways, and nitric oxide signaling—at the tissue level, whereas sc/snRNA‐seq deconvolve these signals into cell‐type‐specific programs across neurons, glia, and vascular cells. Bulk C4A upregulation and inflammatory modules can now be partly reassigned to astrocytes and microglia through co‐expression network analyses,^22^, ^26^ while nasal‐turbinate and organoid scRNA‐seq studies highlight mesenchymal and endothelial compartments as additional immune‐responsive loci.^13^, ^23^, ^24^, ^25^ This re‐annotation moves the field beyond a generic ‘neuroinflammation’ concept toward a model in which coordinated, yet distinct, immune‐related perturbations occur in astrocytes, microglia, endothelial cells, and peripheral‐like stromal cells on a background of primary neuronal/synaptic vulnerability.

## Bipolar Disorder

BD is a psychiatric condition marked by distinct episodes of mood disturbance, including manic or hypomanic episodes and major depressive episodes. Although the precise cause of BD remains unknown, accumulating evidence supports the involvement of genetic factors. Recent studies have identified associations between BD and genetic variants such as *FADS1/2* (fatty acid desaturase 1 and 2) as well as copy number variations involving *RNF216* (ring finger protein 216).^27^, ^28^

Bulk RNA‐seq has contributed to elucidating the link between immune dysfunction and BD. In postmortem human dorsal striatum, transcriptomic profiling identified DEGs—such as *NLRC5*, *S100A12*, *LILRA4*, and *FCGBP*—that were significantly upregulated and enriched in immune and inflammatory pathways.^29^ Bulk RNA‐seq analysis of postmortem brains from individuals with schizophrenia and BD identified 1264 DEGs compared to healthy controls, with many of these genes enriched in biological processes related to the immune system. Although only 28 DEGs were shared between schizophrenia and BD, these genes were predominantly involved in immune system response, immune regulation, and response to stimulus and were all upregulated in BD. Notably, this set included a chemokine‐receptor gene cluster located at 3p21 (*CCDC71*, *CCR1*, and *CCR5*), as well as genes implicated in microglial function (*TREM2*, *TLR1*, *TYROBP*, *C1QA, CD68*, *SERPINA1*, *CD14*, and *AIF1*).^30^ Chen *et al*. reviewed the pathological roles of T cells in BD, primarily synthesizing findings obtained through conventional methods at the time.^31^

sc/snRNA‐seq analyses have further advanced understanding of the link between immune dysfunction and the pathophysiology of BD. In a study integrating scRNA‐seq data from postmortem embryonic and fetal brain tissues with multi‐omics analyses of BD, subpopulations of astrocytes, microglia, and oligodendrocyte progenitor cells associated with BD were identified in the dorsolateral prefrontal cortex.^32^ BD‐associated genes were identified and found to be enriched in biological processes related to inflammation and cellular activation. These findings highlight a significant link between BD and three glial cell types involved in oxidative stress and neuroinflammation. In a study analyzing peripheral blood mononuclear cells (PBMCs) from BD patients before and after treatment with quetiapine and valproate, scRNA‐seq revealed reduced expression of inflammation‐related genes in B cells, along with decreased counts of mast cells and eosinophils.^33^ Additionally, ribosomal peptide synthesis genes were found to be downregulated in both B and T cells following treatment. In a related study focusing on CD8^+^ T cells in BD, the expression of TIM‐3—a key regulator of T‐cell activity closely associated with IL‐6, IL‐1β, and caspase‐3—was significantly elevated compared to controls.^34^ Notably, this upregulation was markedly attenuated after 4 weeks of quetiapine administration.

Overall, bulk RNA‐seq in BD points to robust immune activation—particularly chemokine signaling and microglial‐related pathways—in affected brain regions,^29^, ^30^ whereas sc/snRNA‐seq refine these findings by identifying specific glial and peripheral immune subpopulations that carry these signals.^32^, ^33^, ^34^ Bulk inflammatory modules enriched for microglial and chemokine‐receptor genes can be mapped onto BD‐associated astrocyte, microglial, and oligodendrocyte progenitor subclusters in the prefrontal cortex,^32^ while single‐cell immune profiling of PBMCs reassigns systemic inflammatory signatures to B cells, mast cells, eosinophils, and TIM‐3‐high CD8^+^ T cells.^33^, ^34^ This cell‐type‐resolved perspective indicates that BD immune dysregulation is not uniform, but instead involves distinct glial and adaptive immune compartments.

## Major Depressive Disorder

MDD is a mood disorder characterized by one or more major depressive episodes, which may include persistent low mood, loss of interest or pleasure, feelings of worthlessness, and suicidal ideation. Although its precise etiology remains unknown, immune‐related mechanisms have been implicated in its pathophysiology. For example, a recent study analyzing inflammation‐related molecules in the plasma of patients with MDD, as well as mRNA and protein levels in brain regions such as the hippocampus in a rat model, demonstrated that the Yin‐Yang 1 (YY1)–nuclear factor κB (NF‐κB)–IL‐1β inflammatory pathway plays a critical role in mood disturbances and cognitive dysfunction associated with the disorder.^35^

Bulk RNA‐seq studies have also provided evidence supporting the involvement of immune dysregulation in MDD. In one study analyzing whole blood samples, patients with MDD exhibited elevated expression of genes involved in the IFN‐α/β signaling pathway, including *IRF9*, *OAS1*, and *IFIT3*.^36^ In another study, an analysis of RNA‐seq data from peripheral blood using weighted gene co‐expression network analysis (WGCNA) revealed that gene expression regulation, immune response, and inflammation were associated with suicidal ideation in patients with MDD. In addition, innate immunity, energy metabolism, mitochondrial function, and neurodegeneration were suggested to have broader associations with MDD as a whole.^37^

sc/snRNA‐seq technologies have begun to reveal connections between immune dysfunction and MDD at the cellular level. In an snRNA‐seq study of the dorsolateral prefrontal cortex from postmortem brains of individuals with MDD, a distinct subpopulation of immune oligodendrocytes emerged.^38^ These cells—characterized by the expression of immune‐related genes such as *C3*, *HLA complex*, *P2RY12*, *ADAM28*, *DOCK8*, *LPAR6*, and *ARHGAP24*—have also been recently associated with multiple sclerosis (MS).^39^ Importantly, the immune oligodendrocyte cluster exhibited significantly reduced expression of myelin‐related genes compared to mature oligodendrocytes, suggesting myelination deficits or a maturation arrest of the oligodendrocyte lineage in MDD.^38^ A separate analysis of the same transcriptomic dataset revealed a significantly increased proportion of microglia in MDD.^40^ Further subclustering of the microglial population identified six distinct subclusters enriched in pathways such as the Rap1 signaling pathway, cAMP signaling pathway, TGF‐beta signaling pathway, and ECM–receptor interaction. These processes are involved in neuroinflammation, synaptic plasticity, and neuronal survival, providing further support for the critical role of microglia in the pathogenesis of depression. A peripheral blood single‐cell study also revealed increased numbers of CD14^+^ and CD16^+^ monocytes with high C‐reactive protein levels.^41^ These convergent central and peripheral immune signatures underscore the potential of immune‐based biomarkers for stratifying depression subtypes.

Taken together, bulk RNA‐seq in MDD reveals broad activation of interferon and inflammatory pathways in blood,^36^, ^37^ while single‐cell analyses reannotate these immune modules in terms of specific central and peripheral cell types. snRNA‐seq assigns complement‐ and HLA‐enriched signatures to immune oligodendrocytes with impaired myelin gene expression and to microglial subclusters with distinct inflammatory and signaling profiles in the dorsolateral prefrontal cortex,^38^, ^40^ whereas peripheral scRNA‐seq implicates CD14^+^/CD16^+^ monocytes with heightened immune activity.^41^ This integrated view suggests that MDD‐associated immune dysregulation is instantiated through oligodendrocyte lineage cells and microglia within the brain, in parallel with pro‐inflammatory monocyte populations in the periphery, thereby refining the classical cytokine hypothesis of depression into a more nuanced, cell‐type‐specific model.

## Autism Spectrum Disorder

Autism spectrum disorder (ASD) is a developmental disorder characterized by persistent deficits in social communication and social interaction, along with restricted, repetitive patterns of behavior, interests, or activities. Although ASD is primarily influenced by genetic and prenatal environmental factors, increasing evidence also suggests the involvement of immune dysfunction in its pathophysiology.^42^, ^43^, ^44^ Recent research has suggested a link between ASD symptoms and diHETrE (dihydroxyeicosatrienoic acid), a type of polyunsaturated fatty acid that functions as a key regulator of the immune system.^44^

As in other neuropsychiatric disorders, bulk RNA‐seq has provided evidence supporting the association between immune dysregulation and ASD. RNA‐seq analysis of postmortem superior temporal gyrus tissue revealed elevated expression of stress response and immune activation markers, including heat shock proteins (HSPs) such as HSPA1A/B, DNAJB1/4, and HSPB1/8, as well as HSP‐associated chaperones such as BAG3 and PTGES3. In the same set of samples, RNA‐seq targeting only neuronal cells also revealed increased expression of inflammation‐regulating genes such as *NFKBID* and *AP‐1*, suggesting dysregulated immune signaling in ASD.

sc/snRNA‐seq technologies have also provided evidence for the involvement of immune dysregulation in ASD. In an snRNA‐seq study of the frontal cortex of postmortem brains, reactive forms of microglia and astrocytes were found to be significantly increased in ASD.^45^ Moreover, ASD brains exhibited elevated expression of inflammation‐associated genes, such as *CD47*, *HSPA5*, and *IFI6*, in L2/3 and L5/6 excitatory neurons. A pronounced shift toward an activated inflammatory state, reflected by elevated expression of immune‐related genes such as *HLA‐B*, *P2RX7*, and *IFIT1*, was also observed in glial cell populations, including oligodendrocytes, microglia, astrocytes, and cells forming the BBB. A study focusing on necroptosis‐related genes in snRNA‐seq data from postmortem ASD brains revealed elevated necroptosis activity, particularly in excitatory and inhibitory neurons, as well as in endothelial cells.^46^ Four necroptosis‐related genes—*TICAM1*, *CASP1*, *CAPN1*, and *CHMP4A*—were identified as potential predictors of ASD onset. Additionally, several pathways including oxidative phosphorylation, unfolded protein response, IFN response, and some classic pathways such as p53 and mTORC1 were found to be negatively correlated with ASD. Moreover, immune cell infiltration analyses indicated significantly increased levels of naïve B cells, naïve CD4^+^ T cells, and activated dendritic cells in individuals with ASD. Additional snRNA‐seq of human cortical tissues suggests that synaptic signaling of upper‐layer excitatory neurons and the molecular state of microglia are preferentially impaired in autism.^47^

Together, this integrated view suggests that ASD‐related immune dysregulation is simultaneously instantiated in cortical circuits, glial populations, vascular/endothelial compartments, and peripheral adaptive immunity, thereby refining bulk observation of immune activation into a disturbed, multi‐compartmental immune phenotype.

## Attention‐Deficit and Hyperactivity Disorder

Attention‐deficit/hyperactivity disorder (ADHD) is a neurodevelopmental disorder characterized by persistent symptoms of inattention, impulsivity, and hyperactivity. Although ADHD is known to involve dysfunction of the prefrontal cortex,^48^ and pharmacological treatments targeting this region have been developed, its precise etiology remains unclear. Similar to other psychiatric disorders, ADHD is believed to result from a complex interaction between genetic and environmental factors.^49^

Immune dysregulation in ADHD has been demonstrated using various approaches, including bulk RNA‐seq. In an RNA‐seq study of human PBMCs, co‐expression modules associated with ADHD were identified. These modules included genes involved in immune‐related pathways such as the IL‐17 signaling pathway, TNF signaling pathway, and NF‐κB signaling pathway.^50^

Compared to other psychiatric disorders, sc/snRNA‐seq studies on ADHD are relatively scarce. In one of the few available studies, the effects of ADHD medications—methylphenidate and atomoxetine—were investigated in *Drosophila*.^51^ It was shown that administration of methylphenidate and atomoxetine altered cell–cell interactions mediated by the Toll signaling pathway, a major regulator of innate immunity in *Drosophila*. Moreover, administration was found to induce interactions between monoaminergic neurons and glial cells through the TNF‐α signaling pathway. These findings provide indirect evidence linking immune mechanisms to ADHD.

Thus, bulk blood immune signatures may ultimately map onto specific neuronal–glial interaction networks, but the field still lacks large‐scale human single‐cell datasets required to definitively assign bulk immune modules to particular brain or peripheral cell types in ADHD.

## Alzheimer's Disease

Alzheimer's disease (AD) is the most common form of dementia, characterized by progressive memory loss and cognitive decline. The hallmark pathological features of AD include extracellular amyloid‐β (Aβ) plaques and intracellular neurofibrillary tangles composed of hyperphosphorylated tau protein.^52^ The *APOE* (apolipoprotein E) ε4 allele is the strongest genetic risk factor, associated with elevated cortical Aβ deposition.^53^, ^54^, ^55^ Multiple lines of evidence from experimental, epidemiological, neuropathological, and genetic studies suggest that both innate and adaptive immune activation play a pathological role in the development and progression of AD.^56^

Bulk RNA‐seq analyses have also highlighted the role of immune dysregulation in AD. Multiple studies targeting specific brain regions have demonstrated altered expression of immune‐ and inflammation‐related genes in patients with AD.^57^, ^58^, ^59^, ^60^ For example, expression of *OLR1*, a microglial gene, was found to be altered and showed the strongest association with amyloid plaque burden. Additionally, *CXCR4*, a chemokine receptor involved in the microglial response to neurodegeneration, was upregulated in AD. In a study using laser‐capture microdissection to isolate pathological features for RNA‐seq analysis, Aβ plaques were shown to exhibit upregulation of microglial genes and downregulation of neuronal genes, indicating neuroinflammation and phagocytic activity.^61^

sc/snRNA‐seq have been most actively used in the investigation of neurological rather than psychiatric disorders. Indeed, numerous sc/snRNA‐seq studies focusing on AD, one of the most representative neurodegenerative disorders, have provided various insights into the involvement of immune dysregulation. A study analyzing snRNA‐seq data from postmortem prefrontal cortex tissue investigated gene expression changes in microglia associated with *APOE* genotype.^62^ The results revealed that carriers of the *APOE* ε4 allele exhibited increased expression of inflammation‐related genes, such as *SPP1*, *HSPA1A*, *HSP90AA1*, and *FKBP5*. In an snRNA‐seq study focusing on NEUN^−^OLIG2^−^ nuclei from postmortem brain tissue, a microglial subcluster presumed to respond to Aβ was found to exhibit enriched expression of genes such as *ITGAX*, *LPL*, *GPNMB*, *MYO1E*, and *SPP1*—genes that are also observed in phagocytic or activated microglia in amyloid mouse models.^63^, ^64^, ^65^, ^66^ In contrast, microglia responding to phosphorylated tau were enriched for the expression of homeostatic genes such as *CX3CR1* and *P2RY12*, as well as neuron‐related genes including *GRID2*, *ADGRB3*, and *DPP10*. An snRNA‐seq study using a transgenic mouse model elucidated the mechanistic basis of increased AD risk linked to variants of *INPP5D* (inositol polyphosphate‐5‐phosphatase D), which encodes SHIP‐1 (SH2 domain‐containing inositol 5‐phosphatase 1).^67^ Deletion of SHIP‐1 in microglia led to increased microglial numbers and enhanced recruitment to Aβ plaques. This was accompanied by upregulation of phagocytosis‐related pathways, as well as pathways associated with TREM2/DAP12 signaling and PI3K–AKT–mTOR signaling—both of which are known to promote microglial activation and the transition to a disease‐associated microglial phenotype in AD. Additionally, SHIP‐1 deficiency resulted in downregulation of signaling pathways involved in microglial neuroinflammatory responses, including IL‐6, MAPK, and EGFR1, as well as the TGF‐β signaling pathway, which normally antagonizes microglial activation and responsiveness to Aβ in AD. These findings suggest that SHIP‐1 signaling modulates inflammatory processes in microglia and may act to suppress their protective responses to Aβ plaques.

These sn/scRNA‐seq studies partition these bulk modules into distinct microglial subclusters, including Aβ‐responsive disease‐associated microglia.^63^, ^64^, ^65^, ^66^ Genotype‐stratified snRNA‐seq further links *APOE* ε4 and INPP5D risk alleles to pro‐inflammatory microglial states and altered TREM2/DAP12–PI3K–AKT–mTOR signaling.^62^, ^67^ Thus, what appear as global immune modules in bulk data can now be reinterpreted as arising from specific microglial phenotypes.

## Parkinson's Disease /Parkinson's Disease Dementia /Dementia with Lewy Bodies

Parkinson's disease (PD), Parkinson's disease dementia, and dementia with Lewy bodies (DLB) are neurodegenerative disorders characterized by the accumulation of Lewy bodies—intracellular inclusions primarily composed of aggregated alpha‐synuclein.^68^ While these conditions share pathological features, they differ in clinical presentation and temporal onset of cognitive symptoms. Collectively, these three diseases are referred to as Lewy body diseases (LBD). Previous studies have suggested that immune abnormalities may contribute to the progression and heterogeneity of these diseases.^69^

Bulk RNA‐seq has also revealed associations between immune dysregulation and LBD. In a study examining postmortem brain tissue, transcriptomic analysis of the anterior cingulate cortex from individuals with LBD showed modulation of pathways broadly related to inflammation, including cell activation, inflammatory response, and cytokine production and signaling.^70^ Upregulated DEGs in these pathways included previously reported microglial activation or DAM genes such as *SPP1*, *CSF1*, *TYROBP*, and *TREM2*, as well as immune‐related genes including *CXCL1*, *CXCL8*, *CCL2*, and *CCR5*. Notably, while some disease‐specific transcriptomic changes were observed, the study also demonstrated that core inflammatory pathways are shared between LBD and AD.

sc/snRNA‐seq have also provided evidence for immune involvement in LBD. In one study, scRNA‐seq was performed on CD8^+^ T cells from female patients with early‐ to midstage idiopathic PD. The results revealed elevated expression of cytotoxic effector molecules in effector memory T cells (TEM) and terminally differentiated TEM reexpressing CD45RA (TEMRA) from patients with PD, including *GZMA*, *GZMB*, *GZMH*, *GZMN*, *PRF1*, *NKG7*, *KLRD1*, *KLRG1*, and especially *GNLY*.^71^ The study also demonstrated enhanced pathways related to interactions between lymphocytes and endothelial cells within TEM and TEMRA subsets in PD patients. These findings suggest that T cells may be capable of crossing the BBB or blood–spinal cord barrier, thereby supporting the hypothesis that PD pathogenesis may originate in the periphery and subsequently propagate into the central nervous system. An snRNA‐seq study of postmortem midbrain tissue from individuals with idiopathic PD revealed an increased proportion of microglial cells. These microglia exhibited an inflammatory trajectory characterized by elevated levels of *IL‐1β*, *GPNMB*, and *HSP90AA1*.^72^ In an scRNA‐seq study of cerebrospinal fluid (CSF), CD4^+^ T cells were identified as the most transcriptionally altered immune cell population in patients with clinical DLB and PD.^73^ These cells showed elevated expression of genes associated with enhanced cytokine signaling and activation, including *JAK1*, a kinase essential for cytokine signaling; *CD69*, a T cell activation gene; and *CXCR4*, a chemokine‐receptor gene.

Overall, bulk RNA‐seq in LBD indicates prominent activation of inflammatory and cytokine‐related pathways in affected cortical regions, with enrichment for microglial DAM genes and chemokines shared with AD.^70^ Single‐cell and single‐nucleus datasets refine these signals by assigning them to specific immune subsets across compartments. The diffuse neuroinflammatory signal seen in bulk LBD datasets can be decomposed into a coordinated, multi‐compartment response involving disease‐associated microglia and clonally expanded, tissue‐trafficking T cells, which may together drive or modulate Lewy body pathology.

## Multiple Sclerosis

MS is an autoimmune demyelinating disease of the central nervous system.^74^ Because it can affect any region of the central nervous system, MS is associated with a wide range of neurological symptoms, including psychiatric symptoms. Rather than being caused by a single factor, MS is considered a multifactorial disease resulting from a combination of genetic predisposition and environmental exposures. These include viruses and bacteria such as Epstein–Barr virus, human herpesvirus 6, and *Mycoplasma pneumoniae*, as well as smoking, vitamin deficiencies, dietary factors, and ultraviolet radiation. These elements are thought to interact in complex ways, triggering immune dysregulation that leads to neurological damage.

Bulk RNA‐seq has also revealed immune system abnormalities in MS. In a study analyzing RNA‐seq data from monocytes of MS patients, several well‐known pathways were found to be upregulated, including chemokine signaling, NOD‐like receptor signaling, Jak–STAT signaling, and Toll‐like receptor signaling. These pathways are involved in innate and/or adaptive immune responses.^75^ In addition, the analysis identified novel candidate genes potentially associated with MS, including *RPS4Y1*, *XIST*, *DDX3Y*, *KDM5D*, *KDM6A*, *EIF1AY*, *UTY*, *TXLNGY*, and *PRKY*, as well as enriched pathways such as cell cycle, osteoclast differentiation, ABC transporters, complement and coagulation cascades, Fc gamma receptor‐mediated phagocytosis, and ribosome. In one study using postmortem brain tissue, bulk RNA‐seq was performed on samples categorized by lesion status—normal‐appearing white matter (NAWM) from MS patients, demyelinated white matter lesions (WMLs) from MS patients, and white matter from control donors (CWM).^76^ This analysis identified DEGs between these groups. Biological processes associated with DEGs enriched in NAWM compared to CWM included apoptosis, stress responses, and inflammatory responses, suggesting that pathological changes are already present in NAWM. Furthermore, expression of immediate‐early genes—the first genes transcribed in response to stimuli such as inflammation—was elevated in NAWM relative to CWM. DEGs uniquely enriched in WMLs compared to NAWM were related to epithelial cell apoptosis and cilium assembly and movement, indicating that these processes may occur during or after demyelination or lesion formation. DEGs related to extracellular matrix organization, humoral immune response, leukocyte migration, and complement activation were enriched in both NAWM and WMLs relative to CWM.

sc/snRNA‐seq have also contributed to elucidating the mechanisms of immune dysregulation in MS. In the same study that performed lesion‐specific RNA‐seq analysis, scRNA‐seq was also conducted on macrophages isolated from specific regions of the MS brain, including NAWM, WMLs, and normal‐appearing cortical tissue.^76^ Macrophage clusters enriched in NAWM and WMLs were characterized by high expression of central nervous system‐associated macrophage genes. Those prevalent in NAWM were associated with stress responses and altered oxygen levels, whereas those in WMLs were linked to inflammation and antigen presentation. Macrophages enriched in NAWM also showed elevated expression of HSP genes, immediate‐early genes, and other genes associated with cellular stress. In addition, activated or phagocytic microglia were predominantly found in WMLs and were considered to be involved in demyelination or other myelin‐related processes. In another scRNA‐seq analysis, both blood and CSF samples from MS patients were examined; the results showed that transcriptional diversity was increased in blood, whereas cellular diversity was elevated in the CSF, with an increased proportion of cytotoxic phenotype T helper cells.^77^ These findings suggest site‐specific mechanisms of disease pathogenesis.

Together, bulk RNA‐seq in MS delineates robust immune activation signatures in both monocytes and lesion‐stratified tissues. Single‐cell analyses then map these bulk modules onto specific CNS macrophage and microglial subpopulations.^76^, ^77^ This integration reveals that diffuse inflammatory signatures in bulk data arise from spatially segregated populations of lesion‐associated myeloid cells and compartment‐specific lymphocyte states, providing a cellular basis for the heterogeneity of MS lesions and their progression.

## Anti‐NMDA Receptor Encephalitis

Anti‐N‐methyl‐D‐aspartate receptor (NMDAR) encephalitis (NMDAR‐E) is the most common autoimmune encephalitis and is caused by autoantibodies against NMDAR. It frequently presents with rapidly progressing psychiatric symptoms, abnormal movements, cognitive impairment, and seizures and may be paraneoplastic, often associated with ovarian teratomas. Although most patients respond to immunotherapy, a subset experience poor outcomes. Some patients present only with psychiatric symptoms—so‐called autoimmune psychosis—posing diagnostic challenges in psychiatric settings and highlighting the need for more accessible diagnostic tools.^78^

Evidence of immune dysregulation in NMDAR‐E has also been provided by RNA sequencing technologies, though most studies to date have focused on single‐cell rather than bulk approaches. In a scRNA‐seq study analyzing CSF and PBMCs from patients with NMDAR‐E, a significant increase in B cells—particularly memory B cells—was observed in the CSF.^79^ In these patients, memory B cells in the CSF showed elevated expression of genes related to immunoregulation, such as *TNFRSF13B* and *ITGB1*, whereas peripheral blood B cells exhibited increased expression of antigen presentation genes including *HLA‐DQA2*, *HLA‐DRB5*, and *HLA‐E*. Furthermore, in NMDAR‐E patients, single‐cell B‐cell receptor sequencing revealed that most clonally expanded B cells in the CSF expressed *IGHD*, while peripheral B cells primarily expressed *IGHM*. These findings suggest that CSF B cells in NMDAR‐E undergo class‐switch recombination and may play a role in autoantibody production and dysregulated immune responses. A recent preprint scRNA‐seq study of CSF has further highlighted the role of T cells in NMDAR‐E.^80^ In patients with NMDAR‐E, clonal expansion was observed in both CD4^+^ T and CD8^+^ cells, particularly in CD8^+^ effector memory cells exhibiting enhanced cytotoxic and chemotactic profiles. This study also demonstrated the presence of INF‐responsive B cells in the CSF during the acute phase of NMDAR‐E, as well as a higher proportion of mononuclear phagocytes in the CSF of patients with autoimmune psychosis associated with anti‐NMDAR antibodies.

Although bulk RNA‐seq datasets in NMDA‐E are still limited, single‐cell immune profiling provides a detailed cellular decomposition of the underlying immune response, scRNA‐seq assigns potential bulk immunoregulatory signatures to class‐switched memory B cells and IFN‐response B‐cell states,^79^, ^80^ while peripheral immune activation is mapped to antigen‐presenting B cells and expanded CD4^+^/CD8^+^ TEM populations with cytotoxic and chemotactic programs.^79^, ^80^ Thus, the single‐cell perspective indicates that what would appear as undifferentiated humoral and cellular immune activation in bulk data actually reflect specific B‐cell maturation trajectories and T‐cell effector states that may directly drive autoantibody production and CNS infiltration in NMDAR‐E.

## Integrating Bulk and Single‐Cell Immune Transcriptomics in Neuropsychiatric Disorders

As highlighted throughout this Review, bulk and single‐cell transcriptomic approaches provide complementary perspectives on immune involvement in neuropsychiatric disorders. Bulk RNA‐seq excels at detecting robust, system‐level immune and inflammatory modules across tissues or blood, whereas sc/snRNA‐seq resolve these modules into specific cell types, subclusters, and cellular states. To move beyond descriptive comparisons, an explicit integrative framework is needed. First, bulk RNA‐seq can be used to define reproducible immune and inflammatory modules at the tissue or blood level. Co‐expression and pathway analyses identify signatures such as complement activation, interferon signaling, chemokine networks, microglial activation, or T‐cell effector programs. These modules are typically robust across cohort and platform, but they are agnostic to the exact cellular composition of the samples. Second, sc/snRNA‐seq datasets serve as a reference to assign these bulk modules to specific cell types and disease‐associated subpopulations. By overlaying bulk‐derived gene sets onto single‐cell atlases, one can determine whether a given module is primarily expressed by microglia, astrocytes, oligodendrocytes, endothelial cells, peripheral monocytes, T cells, or B cells, and whether it is confined to particular disease‐associated states. In this way, bulk immune signals are effectively deconvolved into underlying cellular characteristics. Third, integrative computational approaches—such as reference‐based deconvolution, cross‐modal mapping, and network analysis—can formally link bulk and single‐cell signature and quantify cell‐type contributions.^16^, ^17^, ^81^, ^82^ Deconvolution methods can estimate the proportions of major immune and glial cell types in bulk samples, while network integration can identify shared regulatory hubs across bulk and single‐cell layers. Cross‐disorder analyses can then ask whether similar bulk immune modules arise from the same or different cell types across diagnoses, thereby clarifying convergent and divergent immunopathological mechanisms.

Collectively, integrating bulk RNA‐seq and sc/snRNA‐seq along these three steps—bulk module definition, cell‐type assignment, and formal integrative modeling—allows researchers to move from immune signatures as abstract gene lists toward mechanistic models that specify which cells, in which states, and in which tissue compartments, carry these immune programs in each neuropsychiatric disorder.

## Conclusion and Future Perspectives

In this review, we have presented recent findings from sc/snRNA‐seq studies focusing on immune‐related mechanisms across major psychiatric disorders—including schizophrenia, BD, MDD, and neurodevelopmental disorders—as well as neurological disorders such as AD, PD, DLB, MS, and NMDAR‐E. The methodologies and key findings of these studies are summarized in Table S1. As highlighted throughout, single‐cell transcriptomic approaches have begun to uncover the immunopathological processes underlying these disorders.

As demonstrated in a study on schizophrenia by Wu *et al*., sc/snRNA‐seq can detect transcriptional changes that are not identifiable through conventional transcriptomic approaches such as bulk RNA‐seq, underscoring its value.^13^ In addition, sc/snRNA‐seq enable the identification of cell‐type‐specific changes, heterogeneity within a specific cell population, and the detection of rare cell subsets and their transcriptional states. However, compared to bulk RNA‐seq, sc/snRNA‐seq are more expensive, and its data analysis requires specialized expertise due to its complexity. In some cases, isolating specific cell types can also be technically challenging. For these reasons, the emergence of sc/snRNA‐seq has not rendered bulk RNA‐seq obsolete.

In general, RNA‐seq studies on psychiatric disorders are less numerous than those on neurological diseases. Several factors may account for this difference. Neurological disorders often exhibit clear pathological changes, enabling hypothesis‐driven experimental designs, whereas such changes are typically lacking in psychiatric conditions, making study design more difficult. Furthermore, psychiatric diagnoses are based on subjective clinical assessments, resulting in high sample heterogeneity and added complexity in research planning. Psychiatric disorders are also more challenging to model in animals, and obtaining informed consent for human sample collection is often more difficult in psychiatric settings. Consequently, the application of costly technologies such as RNA‐seq has been more limited in psychiatric research. It is noteworthy that immune system involvement has been suggested in both psychiatric and neurological disorders. This commonality implies that immune dysregulation may constitute a shared underlying factor and raises the question of what distinguishes these disease categories despite their immunological overlap. Nevertheless, despite these insights, reported single‐cell data remain insufficient, currently preventing rigorous assessment of cross‐disorder commonalities and differences in the immunopathology of psychiatric disorders. In addition, sc/snRNA‐seq studies face several technical challenges that must be carefully considered. Cell or nucleus isolation procedures can introduce bias by preferentially recovering certain cell types, while low mRNA capture efficiency frequently results in dropout events that obscure true expression patterns. Furthermore, cell‐type annotation often relies on reference datasets or marker genes, which may not fully capture disease‐specific cellular states, leading to ambiguity in classification. These limitations may affect the robustness of biological interpretation, and thus findings should be evaluated in light of these technical constraints.^81^ Continued progress in experimental protocols and computational approaches, such as imputation algorithms for dropout correction, is expected to mitigate these challenges and enhance the reliability of future analyses.^82^

The efficacy of current treatments for many neuropsychiatric disorders remains limited, which may be partly attributed to the underlying heterogeneity within each condition. Continued advancements in RNA‐seq technologies are expected to enable more precise patient stratification, thereby facilitating the implementation of more tailored and effective therapeutic strategies. Although cell‐type‐specific treatments are currently focused primarily on oncology, accumulating knowledge from sc/snRNA‐seq studies on cell‐type‐specific abnormalities in neuropsychiatric disorders may ultimately contribute to the development of novel therapeutic approaches. The integration of other single‐cell technologies—such as single‐cell proteomics and genomics—holds further promise in accelerating this progress. Moreover, emerging methods such as spatial transcriptomics and single‐cell spatial transcriptomics, which allow gene expression analysis while preserving anatomical integrity, are anticipated to significantly advance our understanding of neuropsychiatric diseases.

Taken together, these enriched discussions highlight that psychiatric disorders―schizophrenia, BD, MDD, ASD, and ADHD―harbor distinct yet convergent immune alterations detectable at single‐cell resolution. While neurodegenerative diseases have dominated the single‐cell literature to date, the accumulating psychiatric evidence underscores the urgent need for larger, systematic single‐cell studies in mental illness, which will be indispensable for balanced progress in neuropsychiatric research.

## Author contributions

T.T., M.T., H.T., and M.M. contributed to the concept, overall design, article selection, review, and manuscript preparation. N.I. conceptualized the article and contributed to the overall design.

## Disclosure statement

The authors declare that they have no known competing financial interests or personal relationships that could have appeared to influence the work reported in this paper.

## Supporting information


**Table S1.** Summary of immune‐related findings from single‐cell transcriptomic studies in neuropsychiatric disorders.
